# Deep neural network provides personalized treatment recommendations for *de novo* metastatic breast cancer patients

**DOI:** 10.7150/jca.101293

**Published:** 2024-10-28

**Authors:** Chaofan Li, Yusheng Wang, Haocheng Bai, Mengjie Liu, Yifan Cai, Yu Zhang, Yiwei Jia, Jingkun Qu, Shuqun Zhang, Chong Du

**Affiliations:** 1 The Comprehensive Breast Care Center, The Second Affiliated Hospital of Xi'an Jiaotong University, 157 West Fifth Street, Xi'an, Shaanxi, P. R. China.; 2 Department of Otolaryngology, the Second Affiliated Hospital of Xi'an Jiaotong University, 157 West Fifth Street, Xi'an, Shaanxi, P. R. China.

**Keywords:** dnMBC, surgery, neoadjuvant systemic therapy, deep neural network

## Abstract

**Background:** It has long been controversial whether surgery should be performed for *de novo* metastatic breast cancer (dnMBC). The choice and timing of the primary tumor resection for dnMBC patients need to be individualized, but there was no tool to assist clinicians in decision-making.

**Methods:** A 1:1:2 propensity score matching (PSM) was applied to examine the prognosis of dnMBC patients who underwent neoadjuvant systemic therapy followed by surgery (NS), surgery followed by chemotherapy (SC), and chemotherapy without surgery (CW). Then, two deep feed-forward neural network models were constructed to conduct personalized treatment recommendations.

**Results:** The PSM-adjusted data showed that not all the dnMBC patients could benefit from surgery, and the advantages of NS and SC were different among various subgroups. Patients with stage T1-2, and pathological grade II tumors can be operated on directly, whereas those with stage T3-4, pathological grade III/IV diseases require NS. However, patients with grade I diseases, over 80 years of age, or with brain metastases could not benefit from surgery, regardless of whether they received neoadjuvant systemic therapy. Our deep neural network models exhibited high accuracy on both the train and test sets, one model can assist in deciding whether surgery is requested for dnMBC patient, if the surgery is necessary, another model can determine whether neoadjuvant systemic therapy is needed.

**Conclusion:** This study investigated the prognosis of dnMBC patients, and two artificial intelligence (AI) assisted surgery decision-making models were developed to assist clinicians in delivering precision medicine while improving the survival of dnMBC patients.

## Introduction

*De novo* metastatic breast cancer (dnMBC) is characterized by the spread of BC beyond the breast and regional lymph nodes to distant sites, such as the bone, lung, liver, or brain, at the time of diagnosis. It is a relatively rare condition, affecting approximately 6% of all BC patients 1-3. For these patients, systemic therapy is typically the first choice of treatment since the metastatic invasion can be more lethal than the primary site, but their 5-year overall survival (OS) rate was only 25% 4. It has long been controversial whether patients with dnMBC need to receive surgery. According to previous studies, surgical resection of the primary tumor may even potentially promote the progression of metastases, leading to the deterioration of the disease 5, 6. Consequently, only a few dnMBC patients who had tumor invasion in the chest wall, localized skin disintegration, or other serious complications received local surgery 7. Over the past few decades, the survival rates for patients with dnMBC showed an upward trend owing to the advances in imaging technology, targeted therapy, and immunotherapy, therefore whether surgery or not is getting increasingly debatable 8-12. Several randomized controlled trials (RCTs) indicated locoregional treatment did not appear to improve the OS of patients with dnMBC 13-16, while another clinical trial reached the opposite conclusion 17_._ Numerous retrospective analyses of large cohorts or mono-centric databases have revealed that primary tumor surgery may serve as a therapeutic option for well-selected dnMBC patients 18-26. Therefore, the treatment regime and timing of the primary tumor resection in patients with dnMBC need to be individualized.

Nowadays, artificial intelligence (AI) can help with such problems. AI has emerged as a crucial discipline that offers techniques and tools to explore the vast, high-dimensional, and multi-modal data for biomedical sciences 27-30. Traditional AI algorithms, however, are not robust enough to generate personalized treatment recommendations for patients. While, deep neural networks have been applied in survival prediction and treatment recommendation for cancer patients due to their powerful fitting ability 31-33. Therefore, two deep neural network models were built in this study, one can help us accurately identify dnMBC patients who might benefit from surgery, while the other can help decide the superior sequence of surgery and chemotherapy.

Using the data from the Surveillance Epidemiology and End Results (SEER) database, this study examined and compared the prognoses of dnMBC patients who underwent neoadjuvant systemic therapy followed by surgery (NS), surgery followed by chemotherapy (SC), and chemotherapy without surgery (CW). Two AI-assisted surgery decision-making models were created to help clinicians deliver precision medicine and improve the survival of dnMBC patients.

## Materials and Methods

### Data source and study design

**Figure 1:**
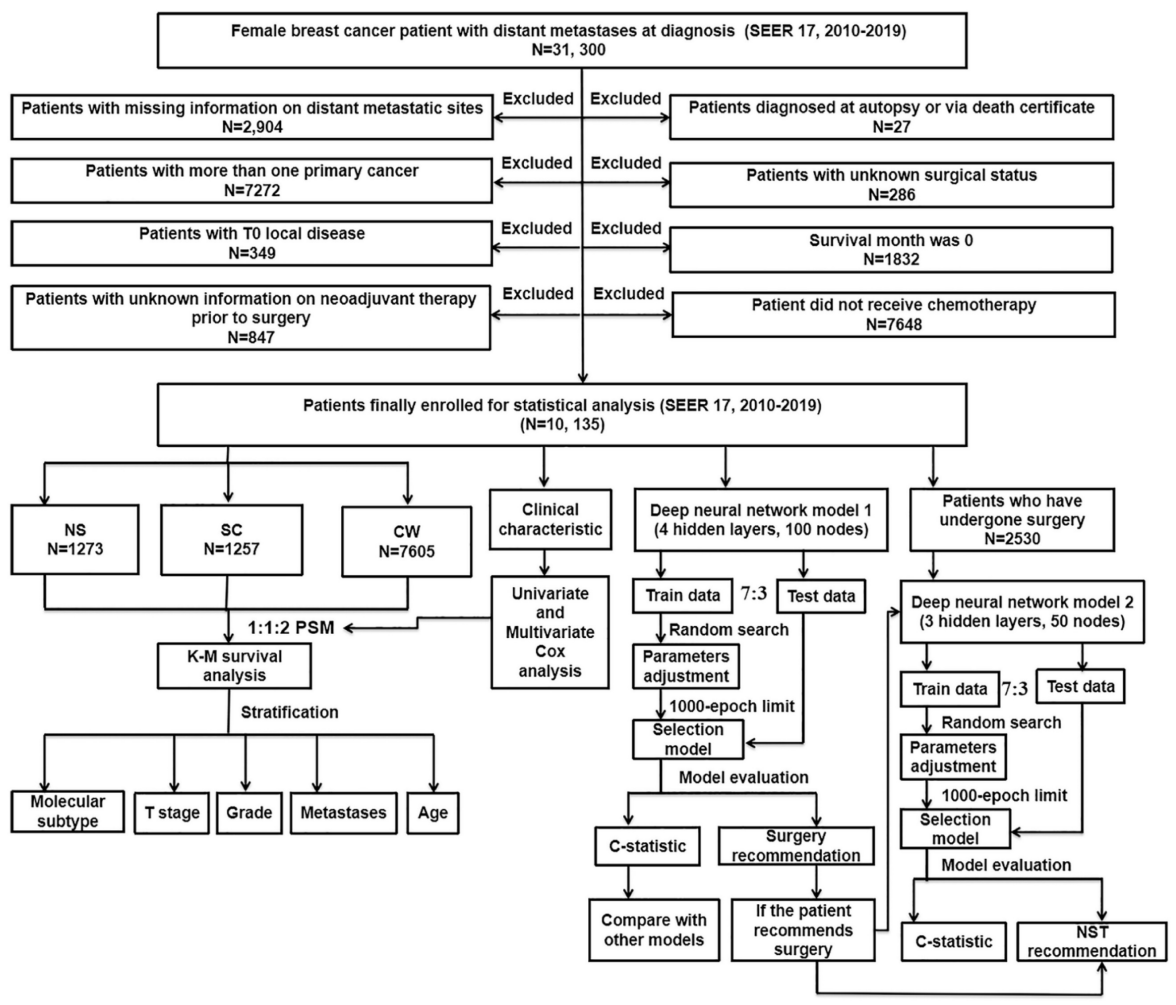
The flowchart detailing the procedure of carrying out the study and statistical analysis. SEER: the Surveillance, Epidemiology, and End Results database; dnMBC: *de novo* metastatic breast cancer; PSM: propensity score matching; Cox: concordance index; K-M: Kaplan-Meier; NS: neoadjuvant systemic therapy followed with surgery, SC: surgery followed with chemotherapy, CW: chemotherapy without surgery; NST: neoadjuvant systemic therapy.

The workflow demonstrating the design of the present study is detailed in **Figure 1**. Our data were collected from the openly accessible SEER database [SEER 17 Regs study data, (changes 2010-2019); version 8.4.0] because it started to collect data about distant metastases in 2010. Only the initial course of therapy, which is defined as the therapeutic regimen administered at initial diagnosis instead of during illness progression or recurrence, is recorded by the SEER database. For this study, data on women with dnMBC were collected. Inclusion criteria: 1) BC was sole cancer developed in each patient; 2) all patients diagnosed with BC showed evidence according to the International Classification of Cancer Diseases Edition III (ICD-O-3) morphological and histopathology diagnosis; 3) all patients with BC progressed to stage IV according to the American Joint Committee on Cancer (AJCC) stage group, 7th editions (2010-2015), SEER combination stage group (2016-2017) and Extent of Disease (EOD) 2018 stage group (2018+) classification. Exclusion criteria: 1) patients diagnosed as BC from death certificate or at autopsy (N = 27); 2) patients with inadequate information on distant metastatic sites (N = 2,904); 3) patients with unknown surgical status (N = 286); 4) patients with more than one primary cancer (N = 7,272); 5) patients with a survival month of 0 (N = 1,832); 6) patients with T0 localized disease (N = 349); 7) patients did not receive chemotherapy (N = 7,648); 8) patients with unknown information on neoadjuvant system therapy prior to surgery (N = 847). Depending on the type of treatment they received, the included patients were then classified into three groups: NS (neoadjuvant systemic therapy followed by surgery), SC (surgery followed by chemotherapy), and CW (chemotherapy without surgery). Follow-up was continued until the death of patients, loss to follow-up, or December 31, 2019.

### Variables

The mode of surgery at the primary site was recorded in the SEER registry under the variable "Surg Prim Site". SEER registry variable "response to neoadjuvant therapy" records the information on the performance and response of neoadjuvant systemic therapy. Clinically, neoadjuvant system therapy refers to the preoperative systemic treatment of patients with resectable BC. Neoadjuvant system therapy described in this database refers to the systemic treatment (hormone/endocrine therapy, chemotherapy, immunotherapy, targeted therapy, or biological therapy) utilized to reduce the tumor size before it was surgically removed (including metastatic cases). The demographic and clinicopathology variables include race, molecular subtype, age at diagnosis, T stage, N stage, histological type, pathological grade, radiotherapy, marital status, median household income (inflation-adjusted), and distant metastases information. The molecular subtype include: hormone receptor-positive /human epidermal growth factor receptor 2-positive (HR+/HER2+), hormone receptor-positive /human epidermal growth factor receptor 2-negative (HR+/HER2-), hormone receptor-negative /human epidermal growth factor receptor 2-positive (HR-/HER2+), and hormone receptor-negative /human epidermal growth factor receptor 2-negative (HR-/HER2-). The pathological grade was typed as grade I (well differentiated), grade II (moderately differentiated), and grade III/IV (poorly differentiated). Overall survival (OS) and breast cancer-specific survival (BCSS) were the main outcomes for our survival analysis. BCSS was determined by deaths attributable to BC. OS, on the other hand, was determined by all causes of death. Both OS and BCSS were determined in the SEER database using cancer registry data and death certificates.

### Statistical analysis

Frequencies and percentages were employed to describe categorical variables. The χ^2^ test or Fisher's exact test was adopted to make between-group comparisons of the categorical data. Univariate Cox regression models were introduced to find out the connection between various demographic and clinicopathology variables and the survival of patients. Further multivariate Cox analysis was conducted to assess the patients' mortality risks and to identify independent prognostic factors. Patients in NS, SC, and CW groups were matched on a 1:1:2 propensity score matching (PSM) to examine the effect of surgical and neoadjuvant system therapy on the prognosis of patients with dnMBC. Due to the number of matched features and the complexity of the classifications, we merged the classifications with similar prognoses according to Cox's results to ensure the quality of the matches. Matching parameters were: method = "nearest", distance = "logit", replace = FALSE, caliper = 0.05. Kaplan-Meier (K-M) survival analyses and log-rank tests were performed on the overall PSM-adjusted data and the data was stratified by molecular subtype, T stage, histological type, grade, and site of metastases. Multiple comparisons were corrected by the Benjamini & Hochberg method. For all statistical calculations, the R programming language (version 4.0.2) was utilized. Statistical significance was defined as a bilateral tail value < 0.05.

### Deep neural network

In this study, two deep feed-forward neural network models were constructed. The models used the deep learning algorithm to predict individual survival risk, which applied deep learning concepts to the Cox proportional risk models by passing the sensitive factor risk function through a multilayer perceptron (and incorporating weight decay regularization, relu activation, batch normalization, dropout, adam, gradient clipping, learning rate adjustment strategy, and other new techniques) to express it in a form that is more adequate to capture the relationship between variables 34. **Supplementary Figure 1** discloses the basic constitution of the models. The inputs of the models were the baseline characteristics of the patients. The hidden layer contained multiple fully-connected layers, weight decay regularisation, dropout, learning rate, etc. The final layer of the model was a single node that was used to estimate the log-risk function of the Cox model. *λ(t)* is the probability that an individual will survive up to time *t* and pass out between time *t* and time* t+t_i_*. The predicted output of the model is a value representing the health risk of the patient.

### Treatment recommendation

Different clinicopathological features and treatment options expose patients to varying degrees of death risks. To determine the specific risk ratio of one treatment option compared to another, the model employs the logarithm of the risk ratio, and this difference in log risk was defined as the recommendation function recij ( x ):

recij (x)=log(λ(t;x|τ=i)/λ(t;x|τ=j))=log(λ0(t)⋅ehi(x)/λ0(t)⋅ehj(x))=hi(x)-hj(x)

For a patient, the treatment recommendation function allows the calculation of the risk value by the model in treatment group i (e.g. surgery) and treatment group j (e.g. no surgery), respectively, and then takes the difference. When the recommendation function recij ( x ) > 0, treatment i (e.g. surgery) results in a higher risk of death than treatment j (e.g. no surgery). Therefore, the patient should receive treatment j (no surgery). The same process is used to recommend neoadjuvant systemic therapy.

Finally, the patients were classified into two groups based on the consistency of the therapy they actually received and the therapy our model recommended: the recommended treatment group and the anti-recommended treatment group. The K-M method was introduced for the examination of OS between different groups, the log-rank test was employed for the comparison of the survival curves, and Cox regression was conducted for the calculation of the hazard ratio (HR).

### Model construction

First, we randomly divided the whole population (N=10, 135) into a train set and a test set according to a 7:3 ratio (**Supplementary Table 1**). The deep neural network model 1 (4 hidden layers, 100 nodes) was constructed based on the train set to predict the prognosis of patients with dnMBC and help to construct a personalized surgical treatment recommendation system. The inputs to the model were independent prognostic factors achieved for Cox analysis: marital status, median household income, race, age, pathological grade, histological type, T stage, surgery, metastatic information, and molecular subtype. Hyperparameter tuning was performed using a random search, then selecting the best-performing model within the limit of 1000 Epochs. To examine the predictive performance of the models, the Harrell C statistic was introduced to evaluate the discriminative power of the network in the train and test datasets, and then it was compared with traditional approaches such as Cox analysis, random survival forest (RSF). The 95% confidence interval (CI) of the C statistic was calculated using the bootstrap method with 1000 resamples.

Next, we randomized the train and test sets in the same 7:3 ratio among patients who underwent surgery (NS group: N = 1,273; SC group: N = 1,257) (**Supplementary Table 2**). The deep neural network model 2 (3 hidden layers, 50 nodes) was developed in the train set to provide personalized treatment recommendations for neoadjuvant system therapy among the patients who received surgery. The treatment with the lower risk value in the model was determined as the recommended therapeutic procedure. Deep neural network models were built on Python 3.11 software and visualized using the "Netron" tool.

## Results

### Clinical characteristics of patients with dnMBC

A total of 10,135 patients with dnMBC diagnosed between 2010 and 2019 were assessed from the SEER database. There were 7,605 patients with CW, 1273 patients with NS, and 1257 patients with SC (**Supplementary Table 3**). Characteristics that were statistically significant among the three groups were age at diagnosis, histological type, molecular subtype, T stage, N stage, grade, median household income (inflation-adjusted), race, marital status, radiotherapy, and distant metastases information (**Supplementary Table 3**).

Patients with NS seemed to be younger than those with CW and SC (NS vs. CW vs. SC: age 60-69: 19.87% vs. 27.48% vs. 27.05%; age 70-79: 8.33% vs. 13.69% vs. 13.76%; age 80+: 1.10% vs. 4.51% and 3.58%). In terms of molecular subtype, a higher proportion of HER2+ was found in the NS group (37.95%) compared with others (CW: 29.57%; SC:28.80%), while a lower proportion of HR+/HER2- subtype was found in the NS group (NS:41.79% vs. CW: 48.56%; SC:49.72%). In terms of stage, patients with NS had relatively higher T stage (T3 and T4: NS: 60.88% vs. CW: 49.02%; SC:38.11%), while patients with CW had relatively early N stage (N2 and N3: CW: 22.47% vs. NS: 41.55%; SC:47.10%). Moreover, patients with NS were more inclined to receive radiotherapy (59.70%), compared to those with CW (28.49%) and SC (39.06%). As for median household income, it was found that patients with SC had relatively poorer household economic status than others. In terms of distant metastases, patients with CW had a lower proportion of single organ metastases (CW vs. NS vs. SC: bone only: 30.11% vs. 42.89% and 40.65%; liver only: 6.96% vs. 11.94% and 12.89%; lung only: 7.76% vs. 12.49% and 11.54%), which means they had relatively more complex distant metastases conditions.

### Univariate and multivariate Cox regression analysis

Our univariate analysis uncovered that therapy, histological type, age at diagnosis, subtype, T stage, N stage, grade, race, marital status, distant metastases information, and median household income were significantly associated with OS and BCSS outcomes (**Table 1**). Notably, we found that radiotherapy did not seem to bring prognostic benefits for dnMBC patients (*p* > 0.05). Further multivariate Cox regression model including all the significant factors from the univariate Cox analysis. The results, showed that compared with patients with CW, patients with NS (OS: HR = 0.500, 95% CI = 0.448-0.557, *p* < 0.001; BCSS: HR = 0.497, 95% CI = 0.443-0.557, *p* < 0.001) and SC (OS: HR = 0.652, 95% CI = 0.589-0.721, *p* < 0.001; BCSS: HR = 0.651, 95%CI = 0.585-0.724, *p* < 0.001) had better OS and BCSS (**Table 2**). We also found that older age, HR-/HER2- subtype, black, other pathological types, unmarried, ≥T3 stage, high grade, low household income, non-single organ, and combined brain metastases were significantly associated with worse OS and BCSS, whereas the N stage was not an independent prognostic factor for patients with dnMBC.

### Benefits of surgical treatment in dnMBC Patients

Based on the results mentioned above, further stratified analysis was necessitated to better compare the difference in the prognosis among the three groups and identify the factors affecting surgical decision-making. A 1:1:2 PSM analysis was carried out to correct the observed imbalance since the baseline characteristics of the three groups varied significantly (**Supplementary Table 3**). Ultimately, 2708 patients were analyzed, and the *p* values for all covariates were greater than 0.05, indicating that their baseline levels are uniform after PSM (**Supplementary Table 4**).

**Figure 2:**
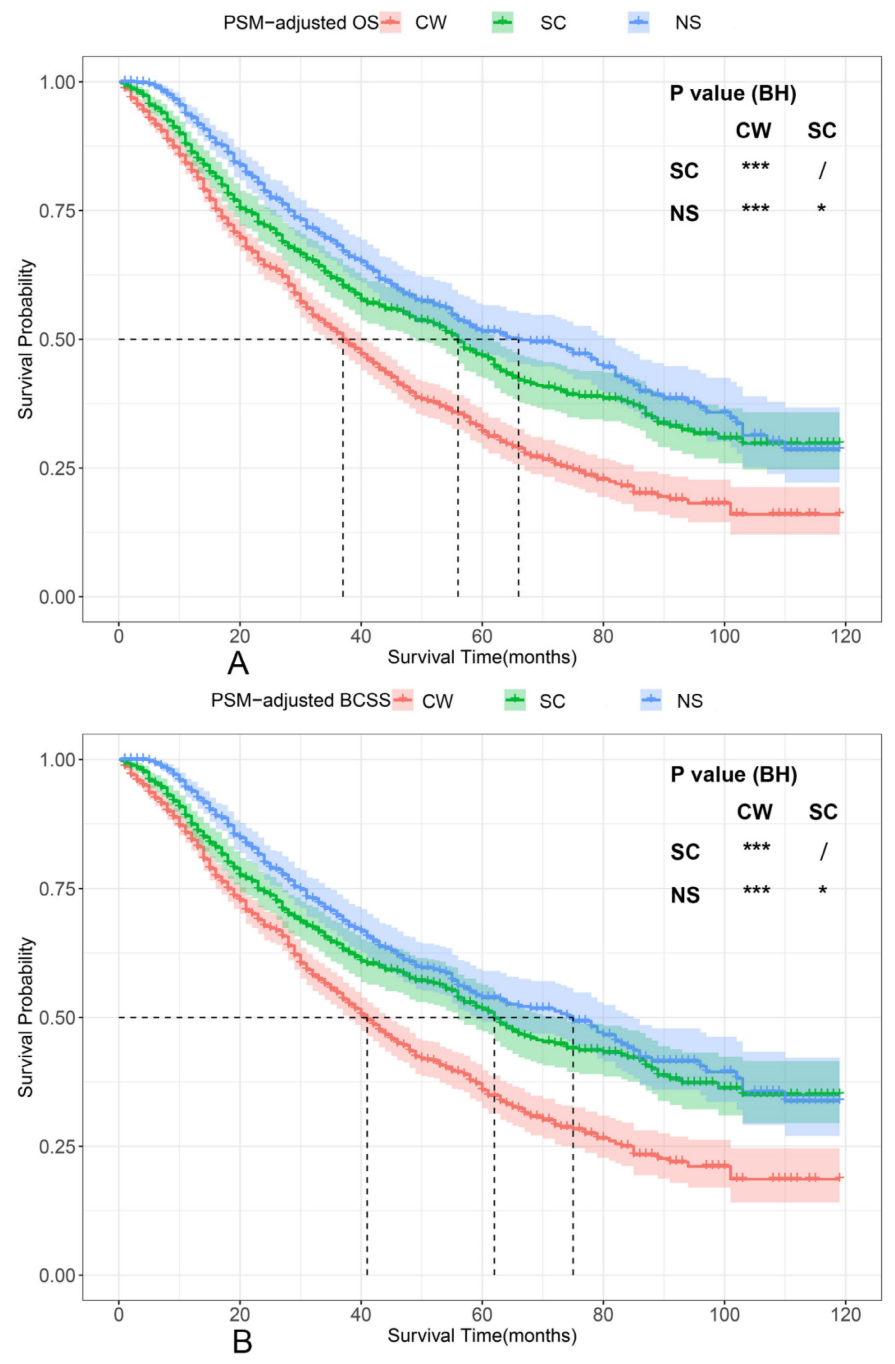
PSM-adjusted OS and BCSS of patients with dnMBC. Kaplan-Meier (K-M) survival analysis: A. OS of patients with dnMBC; B. BCSS of patients with dnMBC; PSM: Propensity score matching; BH: Multiple comparisons were corrected by the Benjamini & Hochberg method; NS: neoadjuvant systemic therapy followed with surgery; SC: surgery followed with chemotherapy; CW: chemotherapy without surgery; dnMBC: *de novo* metastatic breast cancer.

**Figure 3:**
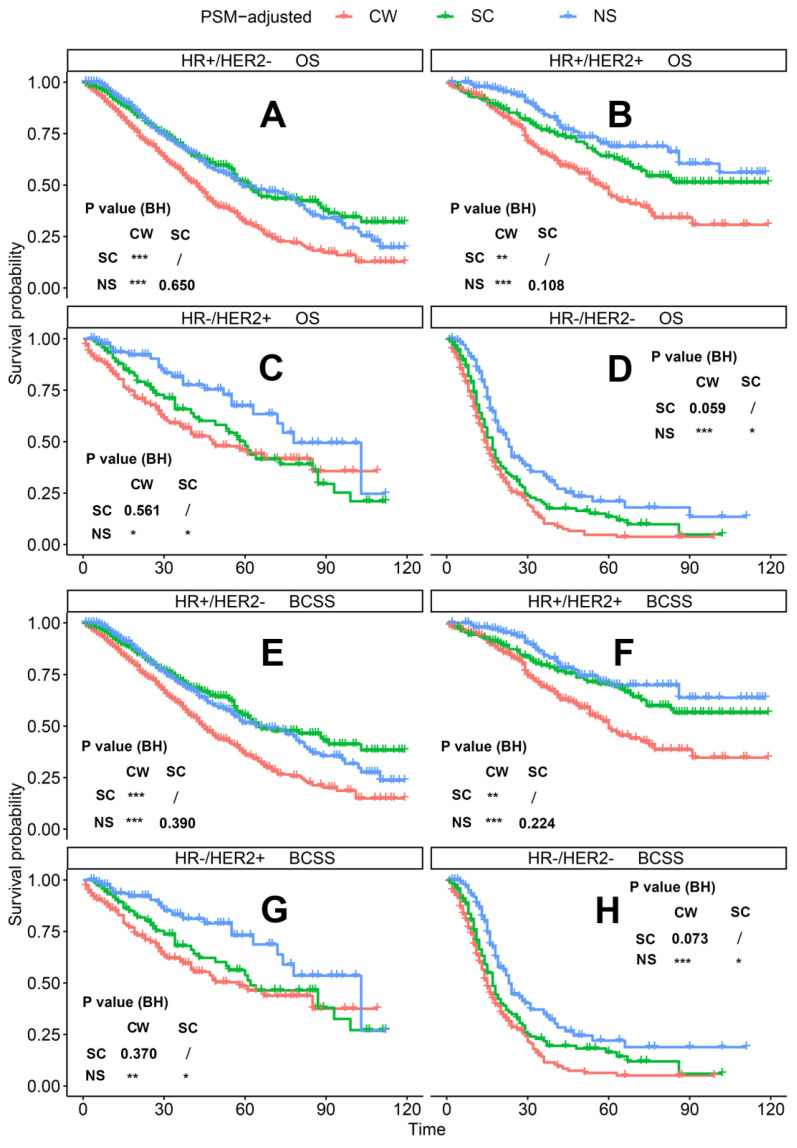
PSM-adjusted OS and BCSS of patients with dnMBC (Stratified by molecular subtype). Kaplan-Meier (K-M) survival analysis: OS of dnMBC patients with (A) HR+/HER2- subtype, (B) HR+/HER2+ subtype, (C) HR-/HER2+ subtype, (D) HR-/HER2- subtype; and BCSS of dnMBC patients with (E) HR+/HER2- subtype, (F) HR+/HER2+ subtype, (G) HR-/HER2+ subtype, (H) HR-/HER2- subtype. OS: overall survival; BCSS: breast cancer-specific survival; dnMBC: *de novo* metastatic breast cancer; HR: hormone receptor; HER2: human epidermal growth factor receptor 2; PSM: Propensity score matching; BH: Multiple comparisons were corrected by the Benjamini & Hochberg method; NS: neoadjuvant systemic therapy followed with surgery; SC: surgery followed with chemotherapy; CW: chemotherapy without surgery.

**Figure 4:**
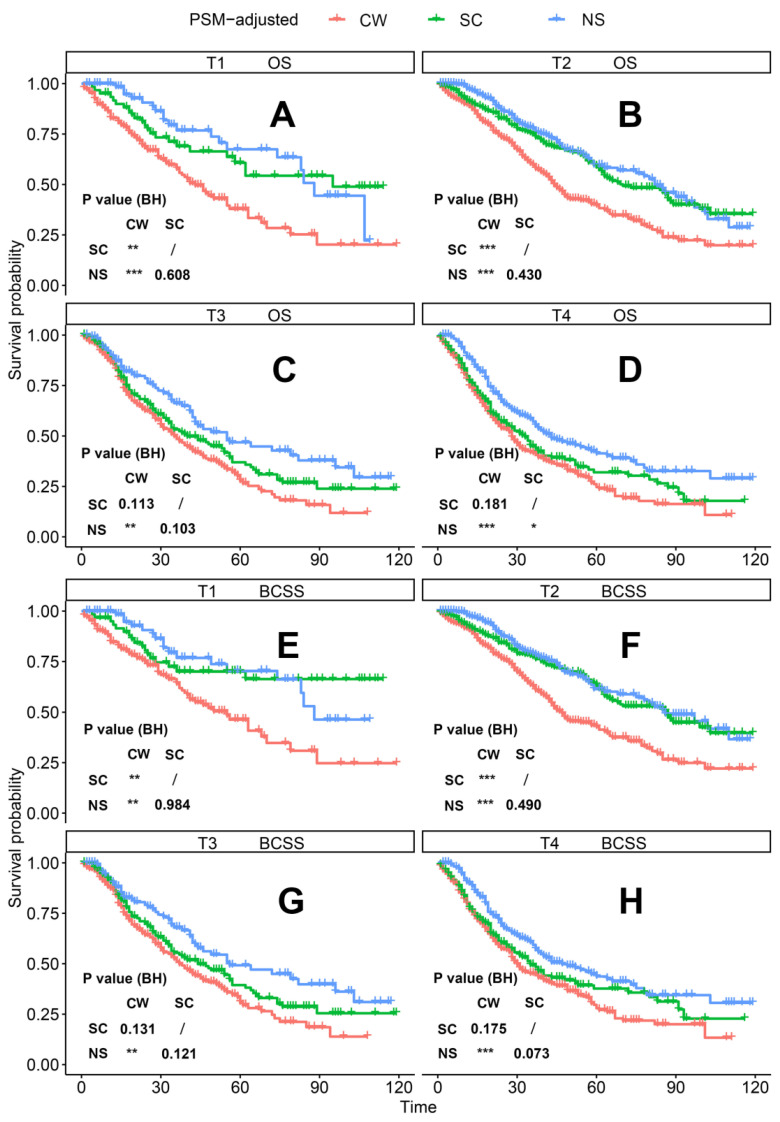
PSM-adjusted OS and BCSS of patients with dnMBC (Stratified by T stage). Kaplan-Meier (K-M) survival analysis: OS of dnMBC patients with (A) T1 stage, (B) T2 stage, (C) T3 stage, (D) T4 stage; and BCSS of dnMBC patients with (E) T1 stage, (F) T2 stage, (G) T3 stage, (H) T4 stage. OS: overall survival; BCSS: breast cancer-specific survival; dnMBC: *de novo* metastatic breast cancer; PSM: Propensity score matching; BH: Multiple comparisons were corrected by the Benjamini & Hochberg method; NS: neoadjuvant systemic therapy followed with surgery; SC: surgery followed with chemotherapy; CW: chemotherapy without surgery.

**Figure 5:**
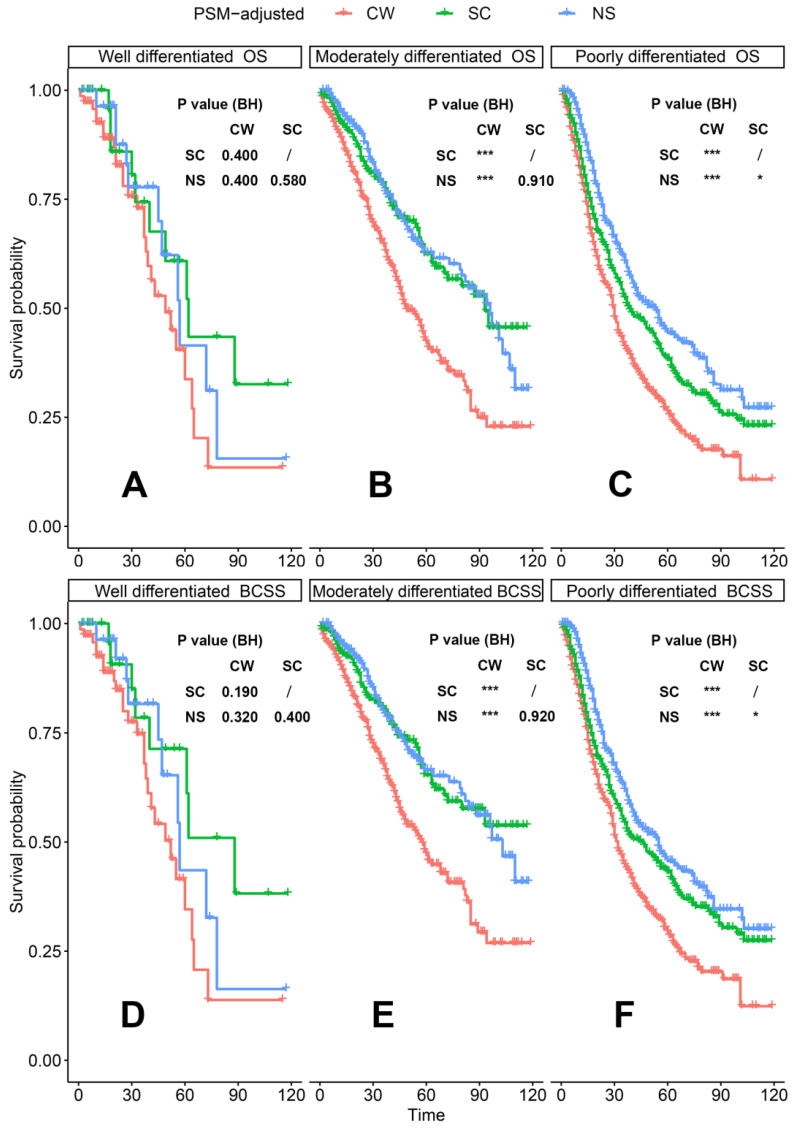
PSM-adjusted OS and BCSS of patients with dnMBC (Stratified by grade). Kaplan-Meier (K-M) survival analysis: OS of dnMBC patients with (A) grade I (well differentiated), (B) grade II (moderately differentiated), (C) grade III/IV (poorly differentiated); BCSS of dnMBC patients with (D) grade I (well differentiated), (E) grade II (moderately differentiated), (F) grade III/IV (poorly differentiated). OS: overall survival; BCSS: breast cancer-specific survival; dnMBC: *de novo* metastatic breast cancer; PSM: Propensity score matching; BH: Multiple comparisons were corrected by the Benjamini & Hochberg method; NS: neoadjuvant systemic therapy followed with surgery; SC: surgery followed with chemotherapy; CW: chemotherapy without surgery.

**Figure 6:**
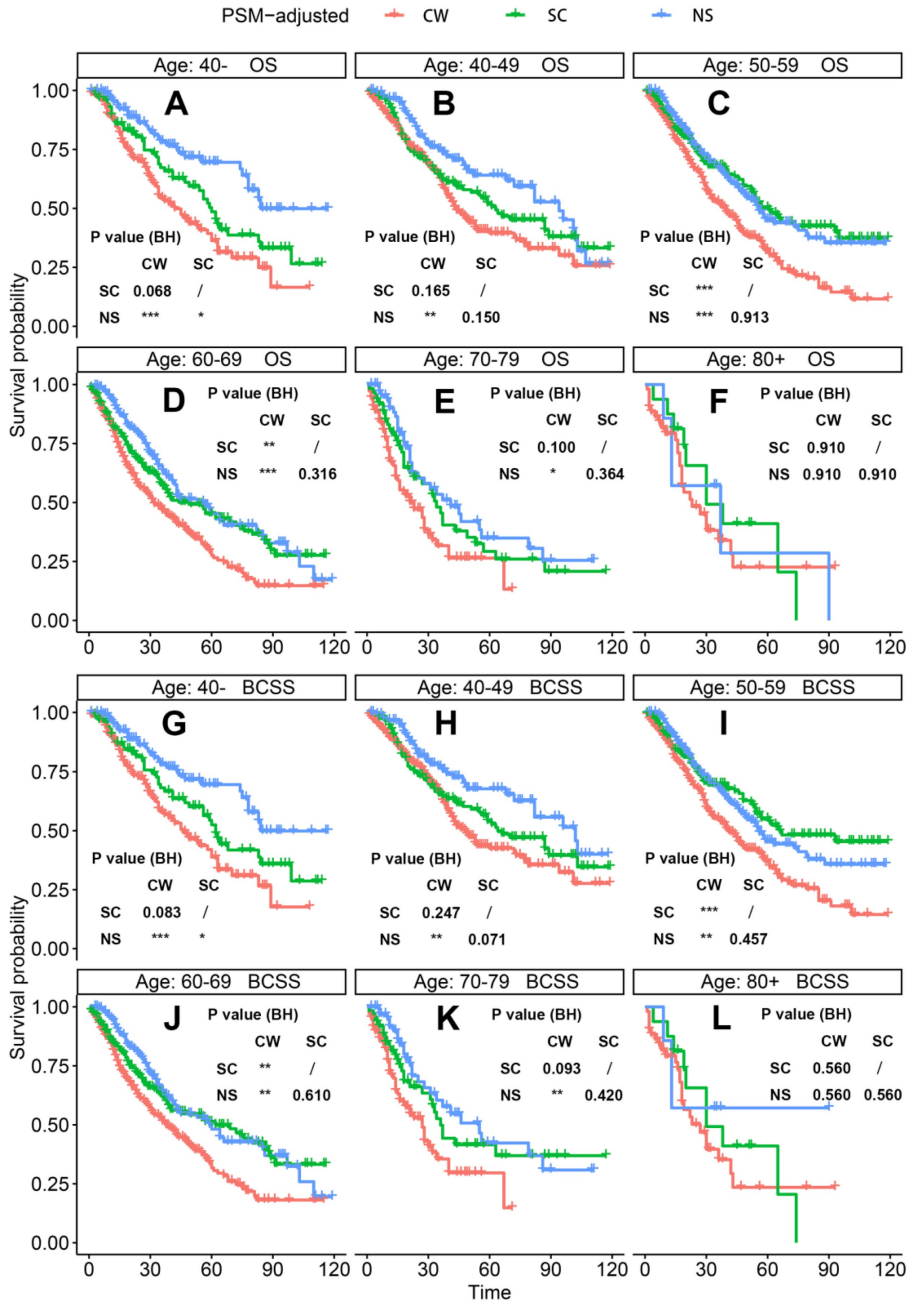
PSM-adjusted OS and BCSS of patients with dnMBC (Stratified by age). Kaplan-Meier (K-M) survival analysis: OS of dnMBC patients aged (A) 40-, (B) 40-49, (C) 50-59, (D) 60-69, (E) 70-79, (F) 80+; BCSS of dnMBC patients aged (G) 40-, (H) 40-49, (I) 50-59, (J) 60-69, (K) 70-79, (L) 80+. OS: overall survival; BCSS: breast cancer-specific survival; dnMBC: *de novo* metastatic breast cancer; PSM: Propensity score matching; BH: Multiple comparisons were corrected by the Benjamini & Hochberg method; NS: neoadjuvant systemic therapy followed with surgery; SC: surgery followed with chemotherapy; CW: chemotherapy without surgery.

**Figure 7:**
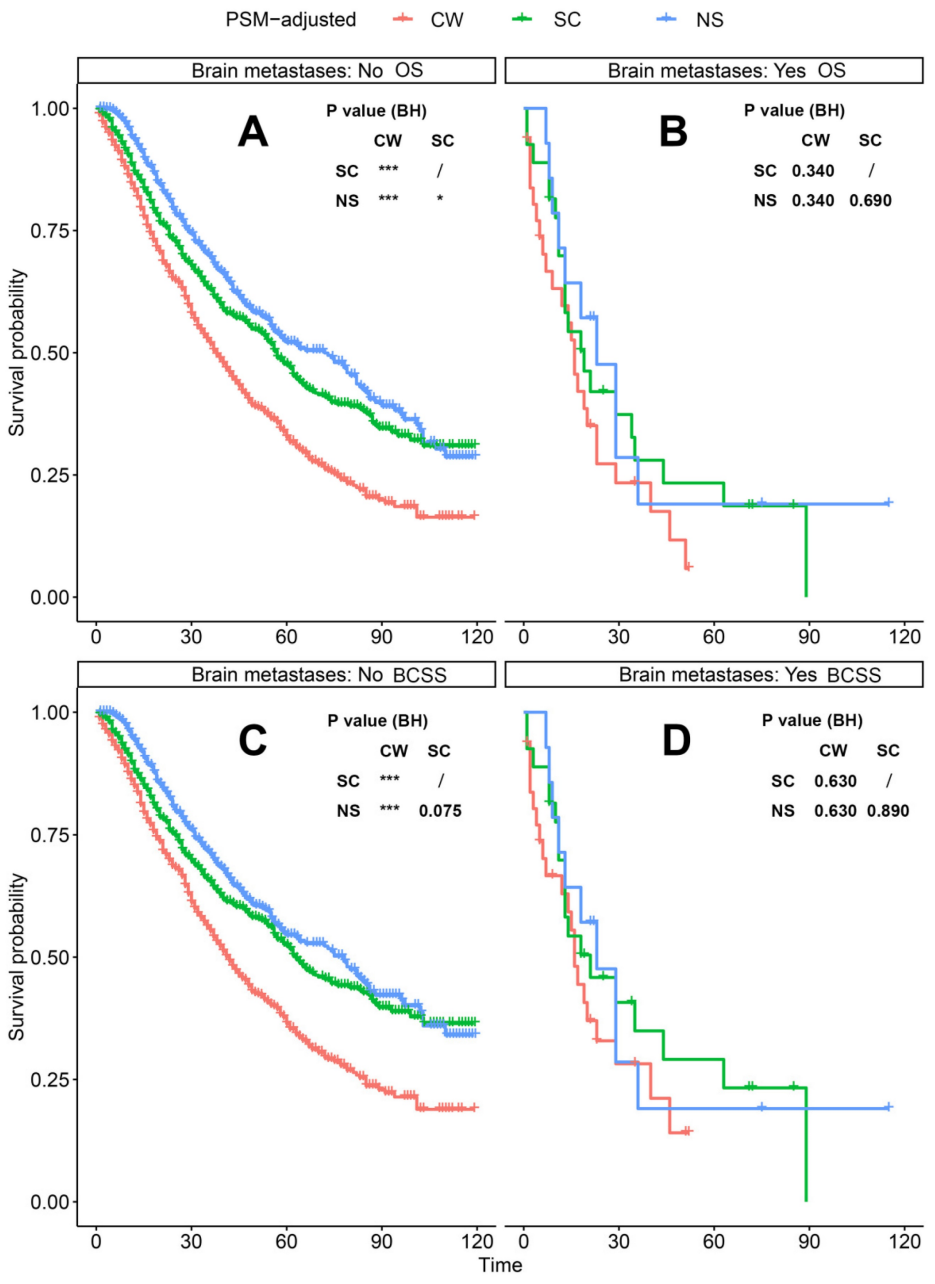
PSM-adjusted OS and BCSS of patients with dnMBC (Stratified by brain metastases). Kaplan-Meier (K-M) survival analysis: A. OS of dnMBC patients without brain metastases; B. OS of dnMBC patients with brain metastases; C. BCSS of dnMBC patients without brain metastases; D. BCSS of dnMBC patients with brain metastases. OS: overall survival; BCSS: breast cancer-specific survival; dnMBC: *de novo* metastatic breast cancer; PSM: Propensity score matching; BH: Multiple comparisons were corrected by the Benjamini & Hochberg method; NS: neoadjuvant systemic therapy followed with surgery; SC: surgery followed with chemotherapy; CW: chemotherapy without surgery.

According to the PSM-adjusted data, surgical intervention significantly improved the OS and BCSS of patients with dnMBC, and those with NS achieved the best prognosis (**Figure 2A** and **2B**). However, the prognoses of dnMBC patients who received the three kinds of treatments varied among different subgroups. Stratified K-M survival analysis showed that, for dnMBC patients with the HR+ subtype, those in the NS and SC groups had better OS and BCSS compared with those in the CW group, while the survival of patients in NS and SC groups showed no significant differences, indicating that surgical intervention could significantly improve the survival of these patients (**Figure 3A-B**, **E-F**). While, for the HR- subtype, only NS could improve the survival of patients (**Figure 3C-D**, **G-H**). In terms of T stage, patients of T1-2 stage can benefit from both NS and SC (**Figure 4A-B**, **E-F**), but only NS could benefit the patients of T3-4 stage (**Figure 4C-D**, **E-F**). In addition to this, the results show that NS was the optimal therapy for patients with poorly differentiated pathology (grade III/IV) (**Figure 5C** and **5F**), NS and SC resulted in the same prognosis for patients with moderately differentiated pathology (grade II) (**Figure 5B** and **5E**), while surgery was not necessary for patients with well differentiated pathology (grade I) (**Figure 5A** and **5D**). For patients of ages < 80, NS could provide survival benefits (**Figure 6A-E** and **6G-K**), while SC could only improve the survival of patients aged 50-69 years (**Figure 6C-D** and **6I-G**). Uniquely, patients aged 80 years or older could not benefit from surgery (**Figure 6F** and **6L**). Finally, we also found that surgery was not suitable for patients with brain metastases (**Figure 7B** and **7D**).

### Establishment and evaluation of the deep neural network models

The results of the stratified K-M survival analysis showed that not all dnMBC patients can benefit from surgery, moreover, the sequence of surgery and chemotherapy did not affect the survival of all patients. Therefore, the therapeutic options of dnMBC required individualized decision-making, and two deep neural network models were built to estimate the OS of dnMBC patients so as to provide recommendations for their treatment. Hyperparameter tuning was performed using a random search, then the best-performing model within the limit of 1000 epochs was chosen. Our deep neural network models 1 and 2 are shown in **Supplementary Figure 2A and 2B**, separately. The C statistics of the train and test datasets were calculated, and compared nomogram, random survival forest (RSF), distant metastases, and subtype with our model (**Table 3**). Overall, our recommendation models for surgery (train set: C statistic=0.811, 95%CI 0.802-0.833; test set: C statistic=0.793, 95%CI 0.781-0.809) and neoadjuvant system therapy (train set: C statistic=0.776, 95%CI 0.754-0.790; test set: C statistic=0.759, 95%CI 0.743-0.782), maintained high accuracy on both the train and test sets. The deep neural network models generated significantly better predictions than others, such as nomogram (train set: C statistic=0.708, 95%CI 0.694-0.717; test set: C statistic=0.702, 95%CI 0.684-0.722), RSF (train set: C statistic=0.724, 95%CI 0.705-0.739; test set: C statistic=0.694, 95%CI 0.678-0.714), subtype (train set: C statistic=0.612, 95%CI 0.595-0.629; test set: C statistic=0.606, 95%CI 0.684-0.722), and distant metastases (train set: C statistic=0.615, 95%CI 0.595-0.634; test set: C statistic=0.608, 95%CI 0.594-0.619).

### Validation of treatment recommendation

**Figure 8:**
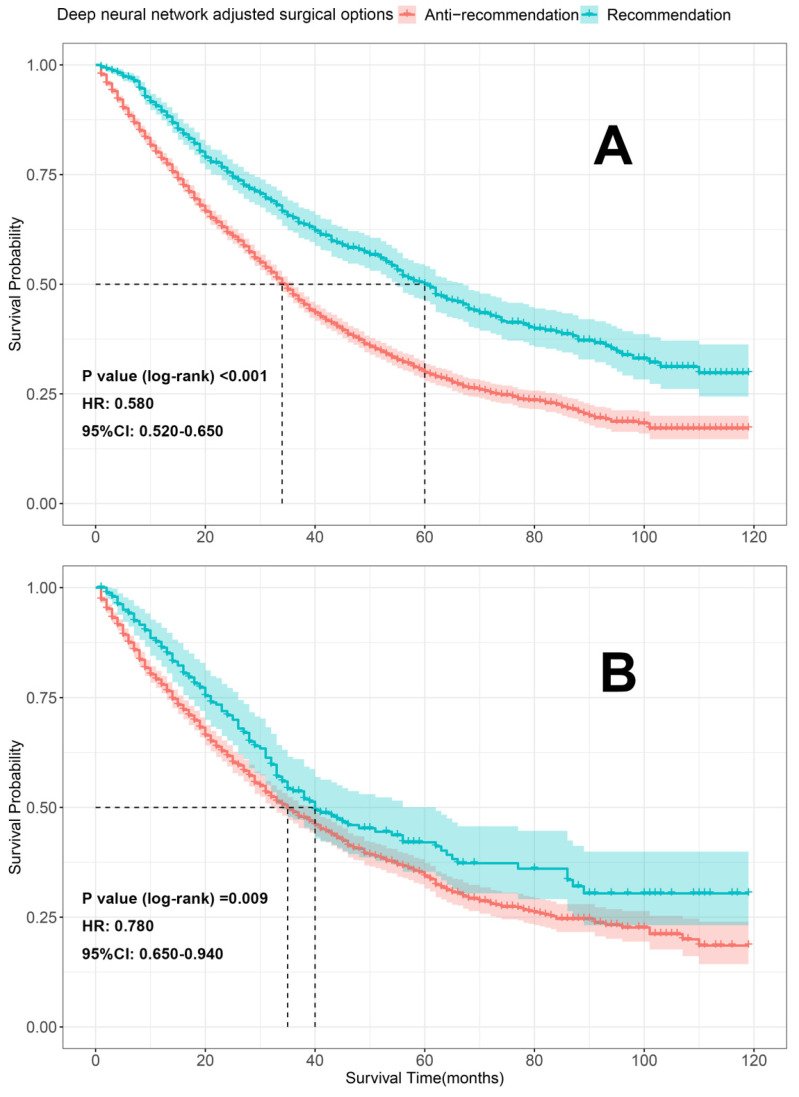
K-M survival analyses comparing the differences in OS between the patients who received the recommendation and anti-recommendation therapy on the train and test sets (deep neural network model 1). A. K-M survival analyses on train set; B. K-M survival analyses on test set; HR: hazard ratio; 95%CI: 95% confidence interval.

**Figure 9:**
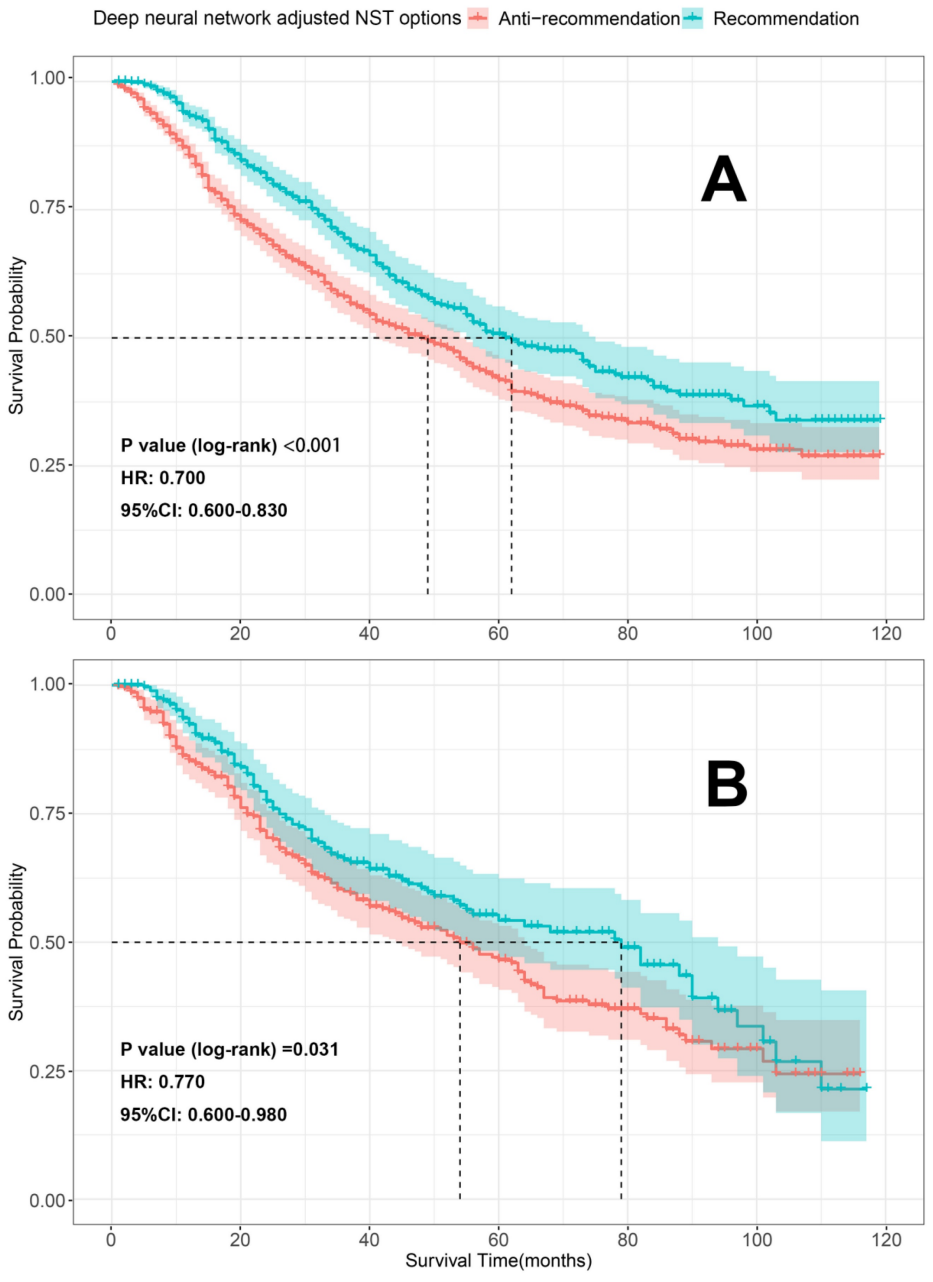
K-M survival analyses comparing the differences in OS between the patients who received the recommendation and anti-recommendation therapy on the train and test sets (deep neural network model 2). A. K-M survival analyses on train set; B. K-M survival analyses on test set; HR: hazard ratio; 95%CI: 95% confidence interval; NST: neoadjuvant systemic therapy.

To further evaluate our models, K-M survival analyses were conducted to compare the differences in OS between the patients who received the recommended (the therapeutic regimen patients actually received consistent with what our model recommended) and anti-recommended therapies (the therapeutic regimen patients actually received were different from what our models recommended). Patients who received the recommended therapies had significantly better OS than those who received anti-recommended therapies in both the train sets [surgery recommendation: HR=0.580, 95% CI 0.520-0.650, *p* < 0.001 (**Figure 8A**); neoadjuvant recommendation: HR=0.700; 95% CI, 0.600-0.830, *p* < 0.001 (**Figure 9A**)] and test sets [surgery recommendation: HR=0.780, 95% CI 0.650-0.940, *p* =0.009 (**Figure 8B**); neoadjuvant recommendation: HR=0.770; 95% CI, 0.600-0.980, *p* =0.031 (**Figure 9B**)]. Overall, our treatment recommendation models can help patients with dnMBC to avoid unnecessary treatments and significantly benefit their survival.

## Discussion

Whether patients with dnMBC need surgery has long been controversial. On the one hand, surgery combined with modern systemic therapies for primary tumors appears to be the perfect companions for eradicating the primary focus, lowering recurrence and metastasis rates^35^, and achieving long-term survival. On the other hand, the systemic administration of highly effective drugs may reduce the benefits of primary tumor resection and, in some cases, make it unnecessary. What appears a lot more challenging and intractable is the timing of surgery, which procedure can generate more benefit when the dnMBC patients need surgery, NS or SC? Several published clinical trials held contradictory views on this. For example, in the ECOG-ACRIN 2108 trial 15, the surgery was performed following a period of systemic therapy, whereas in the MF07-01 17, 36 and ABCSG-28 POSYTIVE trials 16, the surgery was performed directly after diagnosis. They also reached different conclusions, for the MF07-01 study reported the survival benefit gained from surgery, while the other two did not.

This study took a thorough investigation of the SEER database and provided an in-depth view of these controversial issues. The PSM-adjusted results showed that dnMBC patients with NS and SC had a better prognosis than patients with CW, which was basically in line with what Lane et al stated 37. Further stratified survival analysis indicated that not all the dnMBC patients could benefit from surgery, and the advantages of NS and SC were different in some cases. For dnMBC patients with the HR+ subtype, both NS and SC could provide survival benefits, however, only NS could significantly benefit the patients with the HR- subtype. Patients with stage T1-2, and pathological grade II can be operated on directly, whereas for those with stage T3-4, pathological grade III/IV, preoperative neoadjuvant system therapy was indispensable. Patients with grade I, over 80 years of age, or with brain metastases could not benefit from surgery, regardless of whether they received neoadjuvant system therapy. These novel findings enlightened that clinical RCTs can hardly draw similar conclusions due to their constraints of sample size and design protocol, and previously published retrospective studies have not yet progressed to this point. Possible explanations for patients who are not suitable for surgical treatment include the fact that the elderly population is more likely to experience physical and disease deterioration after surgery, thus diminishing the survival benefits gained by surgery. In those patients with brain metastases, the occurrence of impaired consciousness, increased intracranial pressure, and brain herniation make it more important to deal with the metastases immediately rather than the primary site.

The above results may provide some treatment alternatives, but the clinical situation is extensively complicated, for example, should an 80-year-old dnMBC patient with HR+ subtype and stage T3-4 receive surgery or even preoperatively neoadjuvant system therapy? Such questions require more complex and prospective tools to calculate the survival risk for patients receiving various treatment options. Therefore, we created two models by the deep feed-forward neural network to predict the prognosis of dnMBC patients and conduct personalized treatment recommendations. One model can help us decide whether the dnMBC patients need surgery, if the surgery is necessary, another model can tell us whether they need neoadjuvant system therapy. Our models significantly outperform traditional nomogram and RSF in terms of survival prediction, as well as other clinical assessment metrics such as subtype, metastatic status. Previous studies have reported a series of linear models to predict survival in patients with dnMBC 38, 39. However, these models are constrained in the risk factors they can incorporate, and traditional linear models often lack sophistication and therefore the discrimination ability of their models is insufficient. Li et al. constructed a similar deep learning model (DeepMPM) to predict the survival of patients with malignant pleural mesothelioma, with a C-index of 0.7076 (95% CI: 0.7067-0.7086) in the test set 31. Wang et al. also constructed a deep learning survival prediction model, with a C-index of 0.731 in the test set 32. Overall, our model performed better.

Another prominent advantage of our models is their ability to assess the individual risk of mortality for patients receiving different treatments, allowing us to provide patients with individualized treatment and enable precision medicine. Our results showed that OS was significantly higher in patients who received the recommended therapies than those who received anti-recommended therapies, demonstrating the prognostic benefit of making treatment decisions based on the recommendation of our models. Notably, although some studies have employed deep learning models to solve some clinical problems, the majority of them focused on diagnostic applications 40, image interpretation 41-44, or biomarker analysis 45, 46, neglecting the advantages of deep learning in making personalized treatment recommendations. Our study takes advantage of these cutting-edge deep learning techniques and provides an effective tool for the clinical practice of dnMBC.

Despite the encouraging findings we achieved, there were some potential limitations. Firstly, the results of retrospective studies are subject to bias, the selection bias was the most prominent one. Secondly, the SEER database that we used to collect our population data has some inevitable limitations, such as the incomplete collection of treatment information. Finally, the deep neural network models are computationally intensive. Given that deep learning networks operate much like black boxes, their prediction process may be difficult to explain.

## Supplementary Material

Supplementary figures and tables.

## Figures and Tables

**Table 1:** Univariate Cox analysis of characteristics extracted from SEER database

	Univariate Cox analysis					
	OS			BCSS		
	HR	95%CI	P Value	HR	95%CI	P Value
**Therapy**						
CW	reference			reference		
NS	0.479	0.436-0.525	***	0.480	0.435-0.530	***
SC	0.634	0.583-0.689	***	0.634	0.581-0.693	***
**Age at diagnosis**						
<40	reference			reference		
40-49	1.041	0.934-1.160	0.472	1.049	0.936-1.176	0.411
50-59	1.320	1.195-1.457	***	1.316	1.186-1.460	***
60-69	1.409	1.276-1.556	***	1.388	1.250-1.541	***
70-79	1.620	1.449-1.811	***	1.534	1.362-1.727	***
≥80	2.287	1.972-2.652	***	2.180	1.861-2.553	***
**Subtype**						
HR+/HER2-	reference			reference		
HR+/HER2+	0.688	0.636-0.744	***	0.694	0.639-0.754	***
HR-/HER2+	0.973	0.888-1.065	0.547	0.976	0.887-1.075	0.624
HR-/HER2-	2.841	2.644-3.052	***	2.897	2.687-3.125	***
**Race**						
White	reference			reference		
Black	1.414	1.319-1.515	***	1.380	1.282-1.486	***
Others	0.908	0.827-0.996	*	0.882	0.799-0.974	*
**Histological type**						
IDC	reference			reference		
ILC	0.948	0.856-1.050	0.309	0.959	0.861-1.067	0.439
Mixed	0.896	0.795-1.011	0.074	0.917	0.809-1.039	0.175
Others	1.477	1.373-1.589	***	1.434	1.326-1.551	***
**Marital status**						
Married	reference			reference		
Unmarried	1.241	1.175-1.310	***	1.199	1.132-1.270	***
**T Stage**						
T1	reference			reference		
T2	1.045	0.939-1.162	0.423	1.072	0.956-1.201	0.236
T3	1.281	1.144-1.435	***	1.344	1.191-1.516	***
T4	1.572	1.419-1.742	***	1.620	1.451-1.807	***
**N Stage**						
N0	reference			reference		
N1	1.001	0.929-1.079	0.971	1.023	0.945-1.108	0.578
N2	0.969	0.875-1.073	0.544	0.998	0.896-1.111	0.963
N3	1.166	1.066-1.275	***	1.196	1.088-1.315	***
**Grade**						
I; Well differentiated	reference			reference		
II; Moderately differentiated	1.101	0.954-1.270	0.189	1.115	0.957-1.299	0.161
III/IV; Poorly differentiated	1.684	1.465-1.936	***	1.729	1.491-2.005	***
**Median household income (inflation adjusted)**						
<50,000 $	reference			reference		
50,000-59,999 $	0.947	0.859-1.043	0.267	0.961	0.867-1.064	0.442
60,000-69,999 $	0.891	0.818-0.970	**	0.900	0.822-0.985	*
≥70,000 $	0.758	0.696-0.825	***	0.764	0.698-0.836	***
**Radiotherapy**						
None/unknown	reference			reference		
Yes	0.957	0.905-1.013	0.129	0.964	0.908-1.023	0.221
**Distant metastases**						
Bone only	reference			reference		
Liver only	1.143	1.028-1.271	*	1.143	1.021-1.279	*
Lung only	1.384	1.253-1.528	***	1.377	1.239-1.531	***
Brain only	2.319	1.855-2.900	***	2.295	1.808-2.913	***
Bone+Liver	1.766	1.611-1.937	***	1.843	1.674-2.030	***
Bone+Lung	1.552	1.403-1.716	***	1.536	1.380-1.710	***
Bone+Brain	2.217	1.833-2.681	***	2.257	1.848-2.758	***
Bone+Liver+Lung	2.480	2.219-2.772	***	2.542	2.261-2.857	***
Liver+Lung	1.998	1.692-2.357	***	2.060	1.731-2.452	***
Brain+Other	3.162	2.830-3.533	***	3.251	2.894-3.654	***
Other metastases	1.541	1.404-1.691	***	1.524	1.380-1.680	***

Brain+other: cases of brain metastases combined with other metastases except for the bone+brain, e.g. brain+liver etc. Other metastases: other cases of metastases than those listed in the table.

**Table 2:** Multivariate Cox analysis of characteristics extracted from SEER database

	Multivariate Cox analysis					
	OS			BCSS		
	HR	95%CI	P Value	HR	95%CI	P Value
**Therapy**						
CW				**reference**		
NS	0.500	0.448-0.557	***	0.497	0.443-0.557	***
SC	0.652	0.589-0.721	***	0.651	0.585-0.724	***
**Age at diagnosis**						
<40	reference			reference		
40-49	1.082	0.947-1.237	0.246	1.078	0.937-1.240	0.293
50-59	1.278	1.131-1.444	***	1.266	1.114-1.438	***
60-69	1.402	1.236-1.589	***	1.373	1.203-1.566	***
70-79	1.594	1.383-1.838	***	1.514	1.302-1.759	***
≥80	2.424	2.000-2.937	***	2.291	1.867-2.813	***
**Subtype**						
HR+/HER2-				reference		
HR+/HER2+	0.607	0.550-0.670	***	0.601	0.542-0.667	***
HR-/HER2+	0.776	0.691-0.872	***	0.763	0.675-0.863	***
HR-/HER2-	2.569	2.335-2.827	***	2.674	2.419-2.957	***
**Race**						
White	reference			reference		
Black	1.286	1.175-1.409	***	1.249	1.134-1.375	***
Others	0.985	0.877-1.108	0.805	0.962	0.850-1.089	0.536
**Histological type**						
IDC	reference			reference		
ILC	1.103	0.953-1.277	0.190	1.147	0.984-1.337	0.080
Mixed	1.059	0.918-1.223	0.430	1.071	0.921-1.246	0.372
Others	1.206	1.058-1.374	**	1.231	1.074-1.411	**
**Marital status**						
Married	reference			reference		
Unmarried	1.136	1.060-1.217	***	1.122	1.043-1.206	**
**T Stage**						
T1	reference			reference		
T2	1.114	0.980-1.266	0.100	1.145	0.998-1.313	0.053
T3	1.249	1.089-1.433	**	1.318	1.139-1.525	***
T4	1.401	1.231-1.595	***	1.459	1.270-1.675	***
**N Stage**						
N0	reference			reference		
N1	0.954	0.866-1.051	0.340	0.957	0.864-1.060	0.402
N2	1.051	0.926-1.192	0.444	1.069	0.936-1.222	0.325
N3	1.071	0.955-1.202	0.243	1.091	0.965-1.232	0.163
**Grade**						
I; Well differentiated	reference			reference		
II; Moderately differentiated	1.240	1.049-1.466	*	1.248	1.045-1.492	*
III/IV; Poorly differentiated	1.687	1.422-2.000	***	1.718	1.433-2.059	***
**Median household income(inflation adjusted)**						
<50,000$	reference			reference		
50,000-59,999$	0.992	0.880-1.118	0.893	1.040	0.916-1.181	0.547
60,000-69,999$	0.890	0.799-0.990	*	0.915	0.817-1.026	0.130
≥70,000$	0.779	0.699-0.868	***	0.805	0.717-0.903	***
**Radiotherapy**						
None/unknown	reference			reference		
Yes	/	/	/	/	/	/
**Distant metastases**						
Bone only	reference			reference		
Liver only	1.323	1.160-1.510	***	1.343	1.168-1.544	***
Lung only	1.037	0.916-1.174	0.562	1.038	0.910-1.184	0.580
Brain only	1.941	1.430-2.636	***	2.086	1.524-2.855	***
Bone+Liver	1.757	1.559-1.981	***	1.855	1.637-2.103	***
Bone+Lung	1.356	1.189-1.548	***	1.375	1.195-1.582	***
Bone+Brain	2.303	1.778-2.983	***	2.420	1.851-3.164	***
Bone+Liver+Lung	2.464	2.128-2.854	***	2.559	2.193-2.987	***
Liver+Lung	1.628	1.320-2.008	***	1.669	1.339-1.080	***
Brain+Other	2.802	2.410-3.257	***	2.934	2.506-3.434	***
Other metastases	1.365	1.211-1.540	***	1.390	1.223-1.578	***

Brain+other: cases of brain metastases combined with other metastases except for the bone+brain, e.g. brain+liver etc. Other metastases: other cases of metastases than those listed in the table.

**Table 3:** C-statistic of deep neural network on train and test sets

	Train set (95%CI)	Test set (95%CI)
Nomogram	0.708 (0.694-0.717)	0.702 (0.684-0.722)
Subtype	0.612 (0.595-0.629)	0.606 (0.597-0.621)
Distant metastases	0.615 (0.595-0.634)	0.608 (0.594-0.619)
RSF	0.724 (0.705-0.739)	0.694 (0.678-0.714)
Deep neural network model 1	0.811 (0.802-0.833)	0.793 (0.781-0.809)
Deep neural network model 2	0.776 (0.754-0.790)	0.759 (0.743-0.782)
