# Supplementary material for: Deep neural network provides personalized treatment recommendations for de novo metastatic breast cancer patients
Source: J Cancer. 2024 Oct 28;15(20):6668–85. doi: 10.7150/jca.101293 (PMC11632994; doi:10.7150/jca.101293)
Supplement: Supplementary file 1 — Supplementary figures and tables. [file jcav15p6668s1.pdf]

Supplement Figure 1 The basic components of the deep neural network

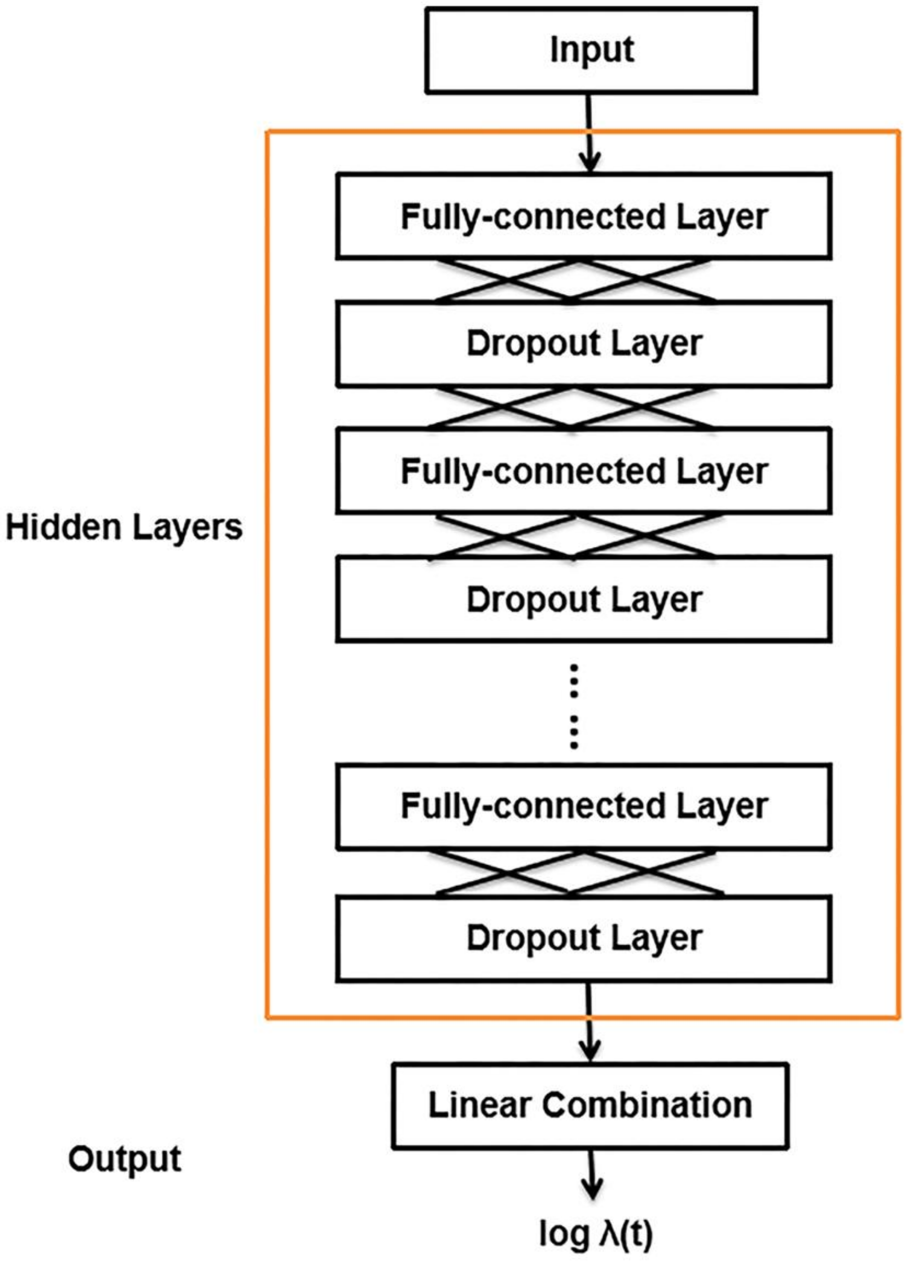

Supplement Figure 2. The deep neural network was visualized using the "Netron" tool.

A. Visualization of deep neural network model 1; B. Visualization of deep neural network model 2.

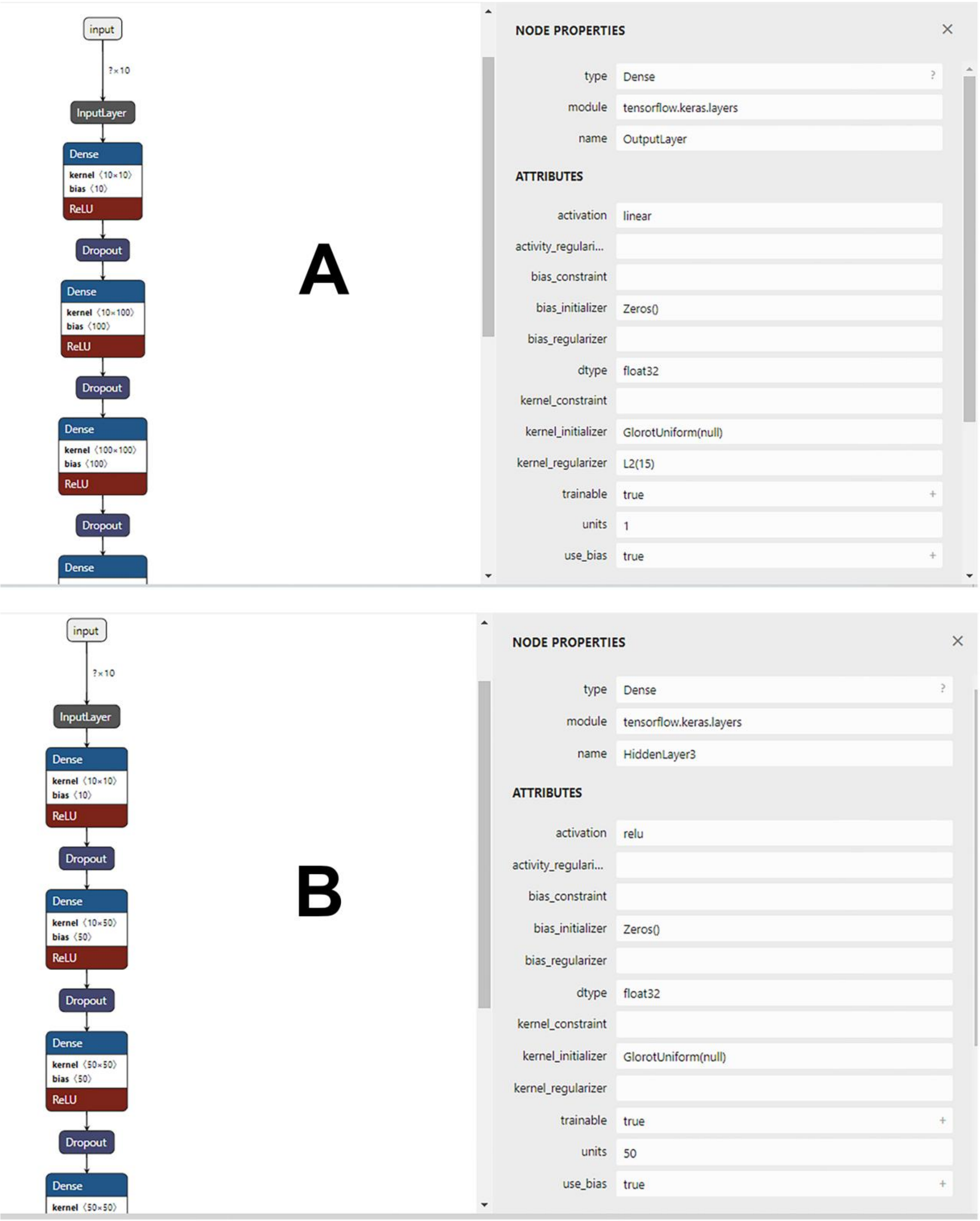

Supplement Table 1. Comparison of patient characteristics in the train and test sets of the deep neural network model 1

	Train set		Test set		P Value
	N=7094	%	N=3041	%	
**Age at diagnosis**					**0.145**
<40	810	11.42%	313	10.29%	
40-49	1232	17.37%	519	17.07%	
50-59	1971	27.78%	885	29.10%	
60-69	1846	26.02%	837	27.52%	
70-79	944	13.31%	376	12.36%	
≥80	291	4.10%	111	3.65%	
**Subtype**					**0.630**
HR+/HER2-	3373	47.55%	1468	48.27%	
HR+/HER2+	1394	19.65%	557	18.32%	
HR-/HER2+	796	11.22%	347	11.41%	
HR-/HER2-	1022	14.41%	452	14.86%	
Unknown	509	7.18%	217	7.14%	
**Race**					**0.566**
White	5221	73.60%	2215	72.84%	
Black	1069	15.07%	490	16.11%	
Others	766	10.80%	318	10.46%	
Unknown	38	0.54%	18	0.59%	
**Histological type**					**0.548**
IDC	5211	73.46%	2231	73.36%	
ILC	581	8.19%	248	8.16%	
Mixed	381	5.37%	146	4.80%	
Others	921	12.98%	416	13.68%	
**Marital status**					**0.681**
Married	3382	47.67%	1449	47.65%	
Unmarried	3396	47.87%	1468	48.27%	
Unknown	316	4.45%	124	4.08%	
**T stage**					**0.347**
T1	686	9.67%	288	9.47%	
T2	2045	28.83%	844	27.75%	
T3	1185	16.70%	479	15.75%	
T4	2290	32.28%	1028	33.80%	
Unknown	888	12.52%	402	13.22%	

**Grade**					**0.673**
I; Well differentiated	369	5.20%	151	4.97%	
II; Moderately differentiated	2251	31.73%	992	32.62%	
III/IV; Poorly differentiated	3077	43.37%	1286	42.29%	
Unknown	1397	19.69%	612	20.12%	
**Median household income (inflation adjusted)**					**0.481**
<50,000$	826	11.64%	365	12.00%	
50,000-59,999$	1100	15.51%	479	15.75%	
60,000-69,999$	2444	34.45%	999	32.85%	
≥70,000$	2724	38.40%	1198	39.39%	
**Surgery**					**0.434**
No	5307	74.81%	2298	75.57%	
Yes	1787	25.19%	743	24.43%	
**Distant metastases**					**0.135**
Bone only	2322	32.73%	1025	33.71%	
Liver only	599	8.44%	244	8.02%	
Lung only	632	8.91%	262	8.62%	
Brain only	77	1.09%	37	1.22%	
Bone+Liver	702	9.90%	263	8.65%	
Bone+Lung	521	7.34%	249	8.19%	
Bone+Brain	117	1.65%	37	1.22%	
Bone+Liver+Lung	354	4.99%	133	4.37%	
Liver+Lung	149	2.10%	69	2.27%	
Brain+Other	360	5.07%	186	6.12%	
Other metastases	1261	17.78%	533	17.53%	

Brain+other: cases of brain metastases combined with other metastases except for the bone+brain, e.g. brain+liver etc. Other metastases: other cases of metastases than those listed in the table.

Supplement Table 2. Comparison of patient characteristics in the train and test sets of the deep neural network model 2

	Train set		Test set		P Value
	N=1787	%	N=743	%	
**Age at diagnosis**					**0.716**
<40	270	15.11%	112	15.07%	
40-49	368	20.59%	155	20.86%	
50-59	488	27.31%	206	27.73%	
60-69	410	22.94%	183	24.63%	
70-79	208	11.64%	71	9.56%	
≥80	43	2.41%	16	2.15%	
**Subtype**					**0.515**
HR+/HER2-	795	44.49%	353	47.51%	
HR+/HER2+	389	21.77%	140	18.84%	
HR-/HER2+	223	12.48%	93	12.52%	
HR-/HER2-	314	17.57%	131	17.63%	
Unknown	66	3.69%	26	3.50%	
**Race**					**0.775**
White	1313	73.48%	542	72.95%	
Black	260	14.55%	115	15.48%	
Others	208	11.64%	85	11.44%	
Unknown	6	0.34%	1	0.13%	
**Histological type**					**0.056**
IDC	1465	81.98%	591	79.54%	
ILC	97	5.43%	51	6.86%	
Mixed	123	6.88%	42	5.65%	
Others	102	5.71%	59	7.94%	
**Marital status**					**0.579**
Married	945	52.88%	376	50.61%	
Unmarried	768	42.98%	335	45.09%	
Unknown	74	4.14%	32	4.31%	
**T stage**					**0.968**
T1	193	10.80%	77	10.36%	
T2	650	36.37%	277	37.28%	
T3	355	19.87%	140	18.84%	
T4	533	29.83%	226	30.42%	
Unknown	56	3.13%	23	3.10%	
**Grade**					**0.572**
I; Well differentiated	79	4.42%	33	4.44%	
II; Moderately differentiated	577	32.29%	261	35.13%	
III/IV; Poorly differentiated	996	55.74%	397	53.43%	

Unknown	135	7.55%	52	7.00%	
**Median household income (inflation adjusted)**					**0.453**
<50,000$	241	13.49%	106	14.27%	
50,000-59,999$	312	17.46%	119	16.02%	
60,000-69,999$	614	34.36%	240	32.30%	
≥70,000$	620	34.70%	278	37.42%	
**Neoadjuvant systemic therapy**					**0.837**
No	885	49.52%	372	50.07%	
Yes	902	50.48%	371	49.93%	
**Distant metastases**					**0.323**
Bone only	730	40.85%	327	44.01%	
Liver only	223	12.48%	91	12.25%	
Lung only	222	12.42%	82	11.04%	
Brain only	23	1.29%	9	1.21%	
Bone+Liver	122	6.83%	38	5.11%	
Bone+Lung	81	4.53%	45	6.06%	
Bone+Brain	13	0.73%	4	0.54%	
Bone+Liver+Lung	44	2.46%	13	1.75%	
Liver+Lung	43	2.41%	10	1.35%	
Brain+Other	38	2.13%	16	2.15%	
Other metastases	248	13.88%	108	14.54%	

Brain+other: cases of brain metastases combined with other metastases except for the bone+brain, e.g. brain+liver etc. Other metastases: other cases of metastases than those listed in the table.

Supplement Table 3: Baseline characteristics of de novo metastatic breast cancer (dnMBC) patients included from SEER data cohort

	CW		NS		SC		P Value
	N=7605	%	N=1273	%	N=1257	%	
**Age at diagnosis**							**<0.001**
<40	741	9.74%	236	18.54%	146	11.61%	
40-49	1228	16.15%	292	22.94%	231	18.38%	
50-59	2162	28.43%	372	29.22%	322	25.62%	
60-69	2090	27.48%	253	19.87%	340	27.05%	
70-79	1041	13.69%	106	8.33%	173	13.76%	
≥80	343	4.51%	14	1.10%	45	3.58%	
**Subtype**							**<0.001**
HR+/HER2-	3693	48.56%	532	41.79%	625	49.72%	
HR+/HER2+	1422	18.70%	307	24.12%	222	17.66%	
HR-/HER2+	827	10.87%	176	13.83%	140	11.14%	
HR-/HER2-	1029	13.53%	227	17.83%	218	17.34%	
Unknown	634	8.34%	40	3.14%	52	4.14%	
**Race**							**0.06**
White	5581	73.39%	924	72.58%	931	74.07%	
Black	1184	15.57%	182	14.30%	193	15.35%	
Others	791	10.40%	163	12.80%	130	10.34%	
Unknown	49	0.64%	4	0.31%	3	0.24%	
**Histological type**							**<0.001**
IDC	5386	70.82%	1072	84.21%	984	78.28%	
ILC	681	8.95%	52	4.08%	96	7.64%	
Mixed	362	4.76%	69	5.42%	96	7.64%	
Others	1176	15.46%	80	6.28%	81	6.44%	
**Marital status**							**<0.001**
Married	3510	46.15%	666	52.32%	655	52.11%	
Unmarried	3761	49.45%	555	43.60%	548	43.60%	
Unknown	334	4.39%	52	4.08%	54	4.30%	
**T stage**							**<0.001**
T1	704	9.26%	112	8.80%	158	12.57%	
T2	1962	25.80%	352	27.65%	575	45.74%	
T3	1169	15.37%	271	21.29%	224	17.82%	
T4	2559	33.65%	504	39.59%	255	20.29%	
Unknown	1211	15.92%	34	2.67%	45	3.58%	
**N stage**							**<0.001**
N0	1426	18.75%	152	11.94%	212	16.87%	
N1	3811	50.11%	574	45.09%	421	33.49%	
N2	597	7.85%	221	17.36%	279	22.20%	
N3	1112	14.62%	308	24.19%	313	24.90%	

Unknown	659	8.67%	18	1.41%	32	2.55%	
**Grade**							**<0.001**
I; Well differentiated	408	5.36%	49	3.85%	63	5.01%	
II; Moderately differentiated	2405	31.62%	428	33.62%	410	32.62%	
III/IV; Poorly differentiated	2970	39.05%	690	54.20%	703	55.93%	
Unknown	1822	23.96%	106	8.33%	81	6.44%	
**Median household income (inflation adjusted)**							**<0.001**
<50,000$	844	11.10%	145	11.39%	202	16.07%	
50,000-59,999$	1148	15.10%	206	16.18%	225	17.90%	
60,000-69,999$	2589	34.04%	462	36.29%	392	31.19%	
≥70,000$	3024	39.76%	460	36.14%	438	34.84%	
**Radiotherapy**							**<0.001**
None/unknown	5438	71.51%	513	40.30%	766	60.94%	
Yes	2167	28.49%	760	59.70%	491	39.06%	
**Distant metastases**						0.00%	**<0.001**
Bone only	2290	30.11%	546	42.89%	511	40.65%	
Liver only	529	6.96%	152	11.94%	162	12.89%	
Lung only	590	7.76%	159	12.49%	145	11.54%	
Brain only	82	1.08%	12	0.94%	20	1.59%	
Bone+Liver	805	10.59%	67	5.26%	93	7.40%	
Bone+Lung	644	8.47%	53	4.16%	73	5.81%	
Bone+Brain	140	1.84%	5	0.39%	12	0.95%	
Bone+Liver+Lung	430	5.65%	25	1.96%	32	2.55%	
Liver+Lung	165	2.17%	23	1.81%	30	2.39%	
Brain+Other	492	6.47%	19	1.49%	35	2.78%	
Other metastases	1438	18.91%	212	16.65%	144	11.46%	

Brain+other: cases of brain metastases combined with other metastases except for the bone+brain, e.g. brain+liver etc. Other metastases: other cases of metastases than those listed in the table.

Supplement Table 4. Comparison of patient characteristics after propensity score matching (PSM)

	CW		NS		SC		P Value
	N=1354	%	N=677	%	N=677	%	
**Age at diagnosis**							**0.944**
<49	451	33.31%	233	34.42%	237	35.01%	
50-69	744	54.95%	369	54.51%	363	53.62%	
≥70	159	11.74%	75	11.08%	77	11.37%	
**Subtype**							**0.167**
HR+/HER2-	693	51.18%	334	49.34%	326	48.15%	
HR+/HER2+	273	20.16%	141	20.83%	140	20.68%	
HR-/HER2+	131	9.68%	85	12.56%	77	11.37%	
HR-/HER2-	196	14.48%	104	15.36%	109	16.10%	
Unknown	55	4.06%	13	1.92%	25	3.69%	
**Race**							**0.642**
White	1009	74.52%	487	71.94%	489	72.23%	
Black	186	13.74%	95	14.03%	101	14.92%	
Others	152	11.23%	93	13.74%	85	12.56%	
Unknown	7	0.52%	2	0.30%	2	0.30%	
**Histological type**							**0.198**
IDC/ILC/Mixed	1315	97.12%	650	96.01%	648	95.72%	
Others	39	2.88%	27	3.99%	29	4.28%	
**Marital status**							**0.637**
Married	702	51.85%	360	53.18%	356	52.58%	
Unmarried	617	45.57%	298	44.02%	296	43.72%	
Unknown	35	2.58%	19	2.81%	25	3.69%	
**T stage**							**0.441**
T1-2	659	48.67%	332	49.04%	330	48.74%	
T3-4	660	48.74%	336	49.63%	329	48.60%	
Unknown	35	2.58%	9	1.33%	18	2.66%	
**Grade**							**0.073**
I; Well differentiated	74	5.47%	29	4.28%	28	4.14%	
II; Moderately differentiated	485	35.82%	235	34.71%	225	33.23%	
III/IV; Poorly differentiated	691	51.03%	364	53.77%	390	57.61%	
Unknown	104	7.68%	49	7.24%	34	5.02%	
**Median household income (inflation adjusted)**							**0.153**
<60,000$	332	24.52%	174	25.70%	193	28.51%	
≥60,000$	1022	75.48%	503	74.30%	484	71.49%	
**Bone metastases**							**0.093**
No	459	33.90%	245	36.19%	248	36.63%	
Yes	895	66.10%	430	63.52%	429	63.37%	
Unknown	0	0.00%	2	0.30%	0	0.00%	
**Liver metastases**							**0.432**

No	1018	75.18%	510	75.33%	500	73.86%	
Yes	330	24.37%	164	24.22%	177	26.14%	
Unknown	6	0.44%	3	0.44%	0	0.00%	
**Lung metastases**							**0.458**
No	1017	75.11%	514	75.92%	499	73.71%	
Yes	332	24.52%	157	23.19%	175	25.85%	
Unknown	5	0.37%	6	0.89%	3	0.44%	
**Brain metastases**							**0.132**
No	1318	97.34%	659	97.34%	648	95.72%	
Yes	32	2.36%	14	2.07%	27	3.99%	
Unknown	4	0.30%	4	0.59%	2	0.30%	
